# Racial differences in setting of implantable cardioverter-defibrillator placement in older adults with heart failure and association with disparate post-implant outcomes

**DOI:** 10.3389/fcvm.2023.1197353

**Published:** 2023-09-01

**Authors:** Ehimare Akhabue, Nathaniel Kuhrt, Poonam Gandhi, Melanie Rua, Uri Shalmon, Aayush Visaria, Larry R Jackson, Soko Setoguchi

**Affiliations:** ^1^ Division of Cardiovascular Diseases and Hypertension, Department of Medicine, Rutgers Robert Wood Johnson Medical School, New Brunswick, NJ, United States; ^2^ Rutgers New Jersey Medical School, Newark, NJ, United States; ^3^ Institute for Health, Health Care Policy and Aging Research, Rutgers University, New Brunswick, NJ, United States; ^4^ Rutgers Robert Wood Johnson Medical School, New Brunswick, NJ, United States; ^5^ Division of Cardiology, Duke Clinical Research Institute, Duke University School of Medicine, Durham, NC, United States

**Keywords:** cardiovascular outcomes, implantable cardioverter-defibrillator, heart failure, primary prevention, racial and ethnic differences

## Abstract

**Background:**

Implantable cardioverter-defibrillator (ICD) placement in heart failure (HF) patients during or early after (≤90 days) unplanned cardiovascular hospitalizations has been associated with poor outcomes. Racial and ethnic differences in this “peri-hospitalization” ICD placement have not been well described.

**Methods:**

Using a 20% random sample of Medicare beneficiaries, we identified older (≥66 years) patients with HF who underwent ICD placement for primary prevention from 2008 to 2018. We investigated racial and ethnic differences in frequency of peri-hospitalization ICD placement using modified Poisson regression. We utilized Kaplan-Meier analyses and Cox regression to investigate the association of peri-hospitalization ICD placement with differences in all-cause mortality and hospitalization (HF, cardiovascular and all-cause) within and between race and ethnicity groups for up to 5-year follow-up.

**Results:**

Among the 61,710 beneficiaries receiving ICDs (35% female, 82% White, 10% Black, 6% Hispanic), 44% were implanted peri-hospitalization. Black [adjusted rate ratio (RR) 95% Confidence Interval (95% CI): 1.16 (1.12, 1.20)] and Hispanic [RR (95% CI): 1.10 (1.06, 1.14)] beneficiaries were more likely than White beneficiaries to have ICD placement peri-hospitalization. Peri-hospitalization ICD placement was associated with an at least 1.5× increased risk of death, 1.5× increased risk of re-hospitalization and 1.7× increased risk of HF hospitalization during 3-year follow-up in fully adjusted models. Although beneficiaries with peri-hospitalization placement had the highest mortality and readmission rates 1- and 3-year post-implant (log-rank *p* < 0.0001), the magnitude of the associated risk did not differ significantly by race and ethnicity (*p* = NS for interaction).

**Conclusions:**

ICD implantation occurring during the peri-hospitalization period was associated with worse prognosis and occurred at higher rates among Black and Hispanic compared to White Medicare beneficiaries with HF during the period under study. The risk associated with peri-hospitalization ICD placement did not differ by race and ethnicity. Future paradigms aimed at enhancing real-world effectiveness of ICD therapy and addressing disparate outcomes should consider timing and setting of ICD placement in HFrEF patients who otherwise meet guideline eligibility.

## Introduction

Implantable cardioverter-defibrillator (ICD) placement is an established interventional therapy for primary prevention of sudden cardiac death (SCD) in patients with heart failure with reduced ejection fraction (HFrEF) (1). Heart Failure (HF) is more common among older (≥65 years) populations and is projected to affect >8 million adults in the United States by 2030 as the population ages (2). Both non-cardiovascular (CV) and CV hospitalizations are common among HF patients (2). Previous studies demonstrated greater peri-procedural complications and increased short-term re-hospitalizations and mortality with ICD placement during or within 90 days of HF hospitalization. As such, ICD implantation in these settings is associated with worse outcomes, despite guideline-based eligibility (3, 4). In general, by current guidelines, ICD placement for primary prevention is recommended in HF patients on optimal guideline directed medical therapy with EF <35% who have expected meaningful survival of >1 year and if an ischemic cardiomyopathy, than ≥40 days post myocardial infarction and ≥90 days post-revascularization (5, 6). There are not firm guidelines otherwise on timing and setting of ICD placement with regards to hospitalization, which not only may affect outcomes but also may have implications for ensuring that overall prognosis related to co-morbidities is adequate enough for ICD placement to confer meaningful survival benefit.

Differences in HF treatment and outcomes by race and ethnicity are well documented. For example, Black populations have higher HFrEF prevalence, hospitalization and mortality rates in addition to lower proportions of eligible patients receiving ICD therapy compared to White populations (2, 7). Previous data have suggested a reduction in racial and ethnic differences in ICD placement in the last decade (8, 9). However, data are lacking on racial and ethnic differences in frequency of ICD placement occurring during or early after unplanned hospitalizations and how these potential differences may contribute to divergent long term outcomes. Given the persistence of disparate HF outcomes by race and ethnicity and to help better understand the real-world effectiveness of ICD therapy for HFrEF (10–13), we sought to address this knowledge gap in a study of older adult Medicare beneficiaries with HF.

## Methods

### Data source and study population

We utilized administrative data from a random 20% sample of all Medicare beneficiaries from 2008 to 2018. Medicare primarily obtains race and ethnicity data from the Social Security Administration, which is self-reported at the time of applying for a social security number. Medicare also utilizes algorithms to enhance the accuracy of some race and ethnicity data by indirect assignment rather than self-report (14). We used the categories specified within the Medicare data based on self-report and these algorithms, including Non-Hispanic White (henceforth White), Non-Hispanic Black (henceforth Black) and Hispanic. For the purposes of this study, due to small populations in the sample prohibiting robust analyses, we aggregated the Asian, Native Hawaiian or Pacific Islander and North American Indian or Alaskan Native categories into a composite “other” category. The Rutgers Biomedical and Health Sciences’ Institutional Review Board approved this study.

#### Inclusion criteria

We identified older adults with HF who had undergone ICD implantation. We applied the following inclusion criteria: (1) ages ≥66 years old at the time of implantation; (2) a HF diagnosis based on *International Classification of Diseases, Ninth-Revision (ICD-9) and Tenth-Revision (ICD-10)* diagnosis codes (ICD-9: 428.XX; ICD-10: I50.XX) prior to ICD implantation; (3) ICD implantation [based on Current Procedural Terminology code (CPT code = 33249)]; (4) ≥12 months of continuous enrollment in Medicare (Part A, B, and D) prior to ICD implantation and (5) non-missing demographic data. This resulted in an initial sample of 103,174 beneficiaries.

#### Exclusion criteria

To focus on beneficiaries receiving an ICD for primary prevention for HFrEF, we then excluded beneficiaries with: (1) inpatient or outpatient diagnosis of cardiac arrest, ventricular tachycardia, ventricular fibrillation or ventricular flutter; (2) inpatient diagnosis of myocardial infarction ≤40 days prior to ICD implantation; (3) revascularization ≤90 days prior to ICD implantation; (4) inpatient or outpatient diagnosis of an arrhythmogenic syndrome (e.g., Brugada, Long QT syndrome); and (5) diagnosis potentially necessitating permanent pacemaker placement during hospitalization. International Classification of Diseases diagnosis and CPT codes for exclusion criteria can be found in Supplementary Table 1 in the supplementary material. The final study cohort was comprised of 61,710 beneficiaries.

### Peri-hospitalization implantable cardioverter-defibrillator placement

Placement of ICDs can be covered by Medicare in both the inpatient and outpatient settings. We defined an inpatient admission that directly followed an emergency room visit as being unplanned. If ICD placement occurred: (1) during an unplanned admission or (2) either during a planned admission or in the ambulatory setting ≤90 days after a prior hospitalization for any cause, we referred to this placement situation as “peri-hospitalization.” ICD placement *not* occurring during an unplanned admission or within 90 days of a previous hospitalization was categorized as “standard” placement for the purposes of this study.

### Outcomes

We investigated the proportion of peri-hospitalization ICD implantations among different racial and ethnic groups (Black, Hispanic, White, “other”). We also investigated long-term post-implant outcomes, including all-cause mortality, HF hospitalization, hospitalization for any CV cause and all-cause hospitalization. Mortality was captured from the Medicare denominator file. Hospitalization type was defined based on diagnosis codes listed in the primary (first) position at discharge as follows: HF hospitalization (ICD-9, 428.xx; ICD-10, I50.xx), CV hospitalization (ICD-9, 390-459; ICD-10, I00-99). Participants were followed for up to 5 years. We do not report data on 5-year outcomes as the findings were generally similar to 3-year outcomes.

### Statistical analysis

We calculated the proportion of ICD placements that occurred peri-hospitalization by race and ethnicity, plotting proportions by year to illustrate crude trends during the study period. To estimate the relative rates of peri-hospitalization ICD placement by race and ethnicity, we used multivariable modified Poisson regression. Multivariable models were constructed sequentially to understand the influence of other factors. Model 1 adjusted for age and sex in addition to race and ethnicity. Model 2 additionally incorporated comorbid diagnoses present in the 12 months preceding ICD placement including: atrial fibrillation, anemia, cancer, chronic obstructive pulmonary disease, diabetes, chronic kidney disease, myocardial infarction, cerebral vascular disease, peripheral vascular disease, and dementia. Model 3 incorporated socio-economic markers, including Medicaid dual-eligibility and information linked to beneficiary zip-code: population density, percent living in poverty, percent owner occupied housing, percent of population that is Black, percent population that is Hispanic and percent completing high school. Model 4 adjusted for health services use preceding ICD placement, including number of outpatient visits and number of prescriptions. Model 5 additionally adjusted for the following in the preceding 12 months: (a) HF therapies, including a beta blocker prescription (metoprolol succinate, carvedilol or bisoprolol), angiotensin-converting enzyme inhibitor, aldosterone receptor blocker or angiotensin-receptor-neprilysin inhibitor therapy, aldosterone receptor antagonist, loop diuretic, hydralazine and isosorbide dinitrate (b) other CV medications including digoxin, warfarin, antiplatelet agent, and non-dihydropyridine calcium channel blockers and (c) whether the implanted device included cardiac resynchronization therapy (CRT, defined by the concurrent presence of CPT code 33225).

We performed Kaplan-Meier analyses to plot survival, HF hospitalization, CV hospitalization and all-cause hospitalization by race and ethnicity and ICD placement status. We compared groups using log-rank tests. The index date was date of ICD placement for mortality and date of discharge for the hospitalization outcomes. For the hospitalization outcomes, beneficiaries were censored at death or end of follow-up. We constructed multivariable Cox proportional hazard regression models to investigate the association of race and peri-hospitalization ICD placement with each outcome. We adjusted for the same covariates as in the modified Poisson regression models except that Cox Models also included adjustment for peri-hospitalization ICD placement. We performed models stratified by race and ethnicity in addition to testing for effect modification in pooled models by including an interaction term between race and ICD placement status. We tested that the proportional hazards assumption was valid using Schoenfeld residuals, assessing each predictor individually by plotting the Schoenfeld residuals against time to check for any patterns.

Two-sided *p*-values <0.05 were deemed statistically significant. All statistical analyses were performed with SAS Enterprise Guide version 8.3 (SAS Institute Inc., Cary, NC).

## Results

Of 61,710 Medicare beneficiaries having an ICD implanted between 2008 and 2018, 81.6% were White, 9.8% were Black, 6.0% were Hispanic and 2.6% were of another race and ethnicity (Table 1). Black beneficiaries had the highest percentage of females (48.1%) than among Hispanic (37.6%) and White (33.3%) beneficiaries. The highest proportions of individuals who were dual-eligible for Medicaid were among Black (24.9%) and Hispanic beneficiaries (41.5%). The highest proportion of atrial fibrillation diagnoses were among White beneficiaries (51.0%), while diabetes was a more common diagnosis among Black (29.9%) and Hispanic beneficiaries (34.7%). There was a higher percentage of loop diuretic prescriptions among Black beneficiaries (73.6%). Black and Hispanic beneficiaries had lower percentages of ICD placements that included CRT (Table 1). Median number of hospitalizations in the 12 months preceding ICD placement was similar by race and ethnicity.

**Table 1:** Demographic and clinical characteristics of population receiving an implantable cardioverter-defibrillator, by race and ethnicity^a^.

	All	White	Black	Hispanic	Other
*N*	61,710	50,334	6,049	3,718	1,609
Peri-hospitalization^b^ ICD	27,094 (43.9%)	21,005 (41.7%)	3,244 (53.6%)	2,031 (54.6%)	814 (50.6%)
During unplanned hospitalization	11,947 (44.1%)	8,816 (42.0%)	1,673 (51.6%)	1,075 (52.9%)	383 (47.1%)
≤90 days post-discharge	15,147 (55.9%)	12,189 (58.0%)	1,571 (48.4%)	956 (47.1%)	431 (52.9%)
Age group, years					
65–74	29,321 (47.5%)	23,244 (46.2%)	3,440 (56.9%)	1,819 (48.9%)	818 (50.8%)
75–84	26,742 (43.3%)	22,224 (44.2%)	2,241 (37.0%)	1,605 (43.2%)	672 (41.8%)
85+	5,647 (9.2%)	4,866 (9.7%)	368 (6.1%)	294 (7.9%)	119 (7.3%)
Female	21,591 (35.0%)	16,742 (33.3%)	2,908 (48.1%)	1,399 (37.6%)	542 (33.7%)
Region					
Northeast	11,902 (19.3%)	9,781 (19.4%)	1,107 (18.3%)	692 (18.6%)	322 (20.0%)
South	26,551 (43.0%)	20,945 (41.6%)	3,531 (58.4%)	1,643 (44.2%)	432 (26.8%)
West	8,305 (13.5%)	6,239 (12.4%)	300 (5.0%)	1,136 (30.6%)	630 (39.2%)
Midwest	14,952 (24.2%)	13,369 (26.6%)	1,111 (18.4%)	247 (6.6%)	225 (14.0%)
Education, less than high school (based on zip code), %	22.1 (13.1)	20.0 (11.3)	29.0 (12.7)	37.6 (20.0)	24.0 (15.0)
Mean percent below poverty (based on zip code)	9.7 (6.8)	8.6 (5.7)	14.0 (8.1)	15.9 (10.1)	11.4 (9.4)
Dual medicare-medicaid eligibility	7,076 (11.5%)	3,407 (6.8%)	1,506 (24.9%)	1,542 (41.5%)	621 (38.6%)
Partial dual medicare-medicaid eligibility	9,637 (15.6%)	4,827 (9.6%)	2,043 (33.8%)	2,042 (54.9%)	725 (45.1%)
Charleston comorbidity index, mean	4.0 (2.0)	3.9 (2.0)	4.3 (2.0)	4.3 (2.0)	4.1 (1.9)
Charleston comorbidity index ≥4	33,415 (54.1%)	26,742 (52.6%)	3,787 (62.6%)	2,218 (59.7%)	938 (58.2%)
Malignancy	17,355 (28.1%)	15,095 (30.0%)	1,264 (20.9%)	673 (18.1%)	323 (20.1%)
Chronic obstructive pulmonary disease	21,405 (34.7%)	17,551 (34.9%)	2,106 (34.8%)	1,273 (34.2%)	475 (29.5%)
Asthma	7,125 (11.5%)	5,347 (10.6%)	977 (16.2%)	573 (15.4%)	228 (14.2%)
Diabetes	14,617 (23.7%)	11,020 (21.9%)	1,807 (29.9%)	1,289 (34.7%)	501 (31.1%)
Chronic kidney disease, stage ≥3	12,913 (20.9%)	10,341 (20.5%)	1,470 (24.3%)	742 (20.0%)	360 (22.4%)
End stage renal disease	2,337 (3.8%)	1,394 (2.8%)	483 (8.0%)	318 (8.6%)	142 (8.8%)
Atrial fibrillation	29,971 (48.6%)	25,662 (51.0%)	2,179 (36.0%)	1,440 (38.7%)	689 (42.8%)
Angina	5,248 (8.5%)	4,362 (8.7%)	392 (6.5%)	338 (9.1%)	156 (9.7%)
Myocardial infarction	10,463 (17.0%)	8,359 (16.6%)	1,051 (17.4%)	742 (20.0%)	311 (19.3%)
Dementia	5,338 (8.7%)	4,107 (8.2%)	643 (10.6%)	433 (11.6%)	155 (9.6%)
Hypertension	56,573 (91.7%)	45,672 (90.7%)	5,865 (97.0%)	3,545 (95.3%)	1,491 (92.7%)
Hyperlipidemia	50,752 (82.2%)	41,460,365 (82.4%)	4,816 (79.6%)	3,150 (84.7%)	1,326 (82.4%)
Smoking	17,562 (28.5%)	14,545 (28.9%)	1,845 (30.5%)	804 (21.6%)	368 (22.9%)
ACEI or ARB	47,224 (76.5%)	38,582 (76.7%)	4,558 (75.3%)	2,891 (77.8%)	1,193 (74.1%)
Aldosterone receptor antagonist	16,733 (27.1%)	13,644 (27.1%)	1,775 (29.3%)	910 (24.5%)	404 (25.1%)
Angiotensin-receptor-neprilysin inhibitor	1,625 (2.6%)	1,363 (2.7%)	146 (2.4%)	76 (2.0%)	40 (2.5%)
Antiarrhythmic	8,071 (13.1%)	7,026 (14.0%)	522 (8.6%)	348 (9.4%)	175 (10.9%)
Non-dihydropyridine CCB	4,221 (6.8%)	3,551 (7.1%)	373 (6.2%)	204 (5.5%)	93 (5.8%)
Loop diuretic	42,441 (68.8%)	34,356 (68.3%)	4,452 (73.6%)	2,565 (69.0%)	1,068 (66.4%)
Beta blocker, any	53,236 (86.3%)	43,449 (86.3%)	5,243 (86.7%)	3,173 (85.3%)	1,371 (85.2%)
Beta blocker, HFrEF guideline-directed	50,888 (82.5%)	41,448 (82.3%)	5,061 (83.7%)	3,060 (82.3%)	1,319 (82.0%)
CRT-D	32,361 (52.4%)	27,421 (54.5%)	2,501 (41.3%)	1,717 (46.2%)	722 (44.9%)
Total number of prescriptions	13 (9, 17)	13 (9, 17)	13 (9, 17)	14 (9, 19)	14 (9, 18)
Outpatient visits prior to ICD	6 (2, 12)	6 (3, 12)	6 (2, 12)	4 (1, 10)	5 (1, 12)
Hospitalizations prior to ICD (median)	1 (0, 2)	1 (0, 1)	1 (0, 2)	1 (0, 2)	1 (0, 2)
Hospitalizations prior to ICD (mean)	1.0 (1.4)	1.0 (1.4)	1.3 (1.6)	1.2 (1.6)	1.0 (1.4)

All are *n* (%), mean (standard deviation) or median (25th, 75th percentile) unless otherwise specified. ACEI or ARB; angiotensin-converting enzyme inhibitor or aldosterone receptor blocker; CCB, calcium channel blocker; CRT-D, cardiac resynchronization therapy defibrillator; HFrEF, heart failure with reduced ejection fraction; ICD, implantable cardioverter-defibrillator.

^a^ Based on data within the 12 months preceding implantable cardioverter-defibrillator implantation.

^b^ Implantable cardioverter-defibrillator placement occurring during an unplanned hospitalization or within 90 days of a previous hospitalization as defined in the primary manuscript text.

**Figure 1:**
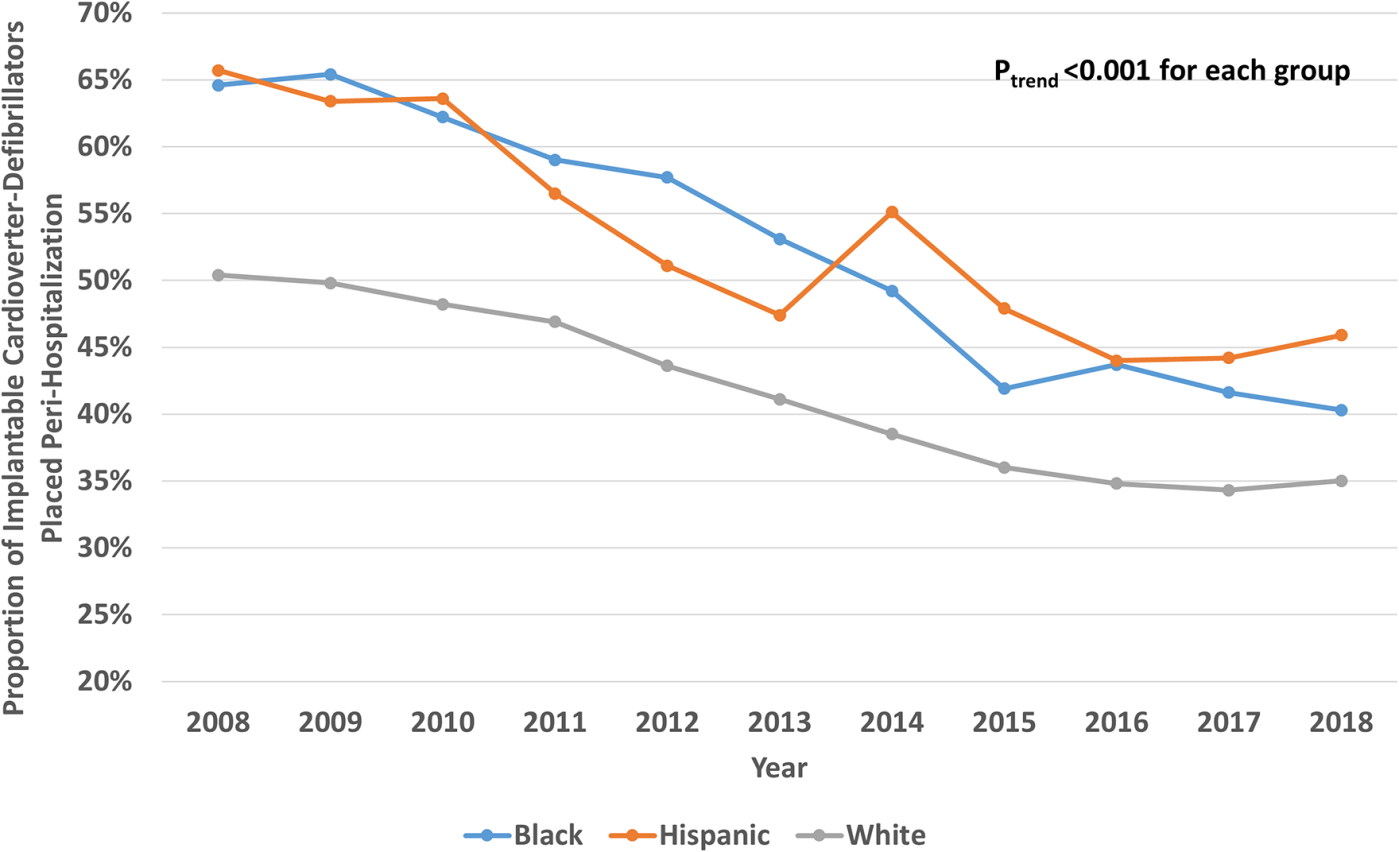
Yearly proportion of implantable cardioverter-defibrillators placed during an unplanned hospitalization or within 90 days of a previous hospitalization (i.e., “Peri-Hospitalization” placement) by Race and Ethnicity.

A higher overall percentage of Black (53.6%, *n* = 3,244/6,049) and Hispanic (54.6%, *n* = 2,031/3,718) beneficiaries received an ICD peri-hospitalization—defined as occurring during an unplanned admission or ≤90 days after a prior hospitalization for any cause—compared with White beneficiaries (41.7%, *n* = 21,005/50,334). The majority of the peri-hospitalization placements among Black and Hispanic beneficiaries occurred during an unplanned hospitalization whereas among White beneficiaries the majority occurred ≤90 days after discharge from a previous hospitalization (Table 1). In general, beneficiaries with peri-hospitalization placement had a higher prevalence of comorbid conditions (Table 2). The crude rate of peri-hospitalization ICD implants declined over time in all race and ethnicity groups (Figure 1, *p* for trend <0.001 for all races) but the lower rates in White beneficiaries compared to Black and Hispanic groups seem to have persisted throughout the study period (Figure 1). In fully adjusted modified Poisson regression models, Black beneficiaries were 16% [Table 3: Risk ratio 1.16 (1.12, 1.20)] and Hispanic 10% [RR 1.10 (1.06, 1.14)] more likely than White beneficiaries to have had their ICD placed peri-hospitalization. In fully adjusted models, female sex was also associated with a minimally increased rate of peri-hospitalization ICD placement [RR: 1.05 (1.03, 1.07)].

**Table 2:** Demographic and clinical characteristics of population receiving an implantable cardioverter-defibrillator, by race, ethnicity and placement category.

	White		Black		Hispanic	
	Standard	Peri-hospitalization^a^	Standard	Peri-hospitalization^a^	Standard	Peri-hospitalization^a^
*N*	29,329	21,005	2,805	3,244	1,687	2,031
Age group, years						
65–74	14,321 (48.8%)	8,923 (42.5%)	1,652 (58.9%)	1,788 (55.1%)	887 (52.6%)	932 (45.9%)
75–84	12,695 (43.3%)	9,529 (45.4%)	1,021 (36.4%)	1,220 (37.6%)	690 (40.9%)	915 (45.1%)
85+	2,313 (7.9%)	2,553 (12.2%)	132 (4.7%)	236 (7.3%)	110 (6.5%)	184 (9.1%)
Female	9,631 (32.8%)	7,111 (33.9%)	1,332 (47.5%)	1,576 (48.6%)	600 (35.6%)	799 (39.3%)
Region						
Northeast	5,085 (17.3%)	4,696 (22.4%)	416 (14.8%)	691 (21.3%)	284 (16.8%)	408 (20.1%)
South	12,745 (43.5%)	8,200 (39.0%)	1,799 (64.1%)	1,732 (53.4%)	755 (44.8%)	888 (43.7%)
West	3,617 (12.3%)	2,622 (12.5%)	123 (4.4%)	177 (5.5%)	532 (31.5%)	604 (29.7%)
Midwest	7,882 (26.9%)	5,487 (26.1%)	467 (16.6%)	644 (19.9%)	116 (6.9%)	131 (6.5%)
Education, less than high school (based on zip code), %	19.7% (11.2)	20.5% (11.5)	28.1% (12.6)	29.7% (12.7)	35.6% (20.0)	39.2% (19.9%)
Mean percent below poverty (based on zip code)	8.6% (5.6)	8.7% (5.8)	13.6% (7.9)	14.4% (8.3)	15.1% (9.8)	16.5% (10.4)
Dual medicare-medicaid eligibility	1,577 (5.4%)	1,830 (8.7%)	568 (20.2%)	938 (28.9%)	601 (35.6%)	941 (46.3%)
Charleston comorbidity index, mean	3.7 (1.9)	4.3 (2.1)	4.0 (1.9)	4.7 (2.0)	3.8 (1.9)	4.7 (2.1)
Charleston comorbidity index ≥4	13,688 (46.7%)	12,784 (60.9%)	1,563 (55.7%)	2,224 (68.6%)	836 (49.6%)	1,382 (68.0%)
Malignancy	8,845 (30.2%)	6,250 (29.8%)	572 (20.4%)	692 (21.3%)	272 (16.1%)	401 (19.7%)
Chronic obstructive pulmonary disease	8,347 (28.5%)	9,204 (43.8%)	736 (26.2%)	1,370 (42.2%)	412 (24.4%)	861 (42.4%)
Asthma	2,628 (9.0%)	2,719 (12.9%)	344 (12.3%)	633 (19.5%)	169 (10.0%)	404 (19.9%)
Diabetes	5,494 (18.7%)	5,526 (26.3%)	738 (26.3%)	1,069 (33.0%)	492 (29.2%)	797 (39.2%)
Chronic kidney disease, stage ≥3	4,656 (15.9%)	5,685 (27.1%)	564 (20.1%)	906 (27.9%)	246 (14.6%)	496 (24.4%)
End stage renal disease	490 (1.7%)	904 (4.3%)	161 (5.7%)	322 (9.9%)	89 (5.3%)	229 (11.3%)
Atrial fibrillation	12,960 (44.2%)	12,703 (60.5%)	781 (27.8%)	1,398 (43.1%)	517 (30.6%)	923 (45.4%)
Myocardial infarction	3,665 (12.5%)	4,694 (22.3%)	356 (12.7%)	695 (21.4%)	236 (14.0%)	506 (24.9%)
Dementia	1,628 (5.6%)	2,479 (11.8%)	174 (6.2%)	469 (14.5%)	138 (8.2%)	295 (14.5%)
Hypertension	25,786 (87.9%)	19,886 (94.7%)	2,677 (95.4%)	3,188 (98.3)	1,572 (93.2%)	1,973 (97.1%)
Hyperlipidemia	23,612 (80.5%)	17,848 (85.0%)	2,180 (77.7%)	2,636 (81.3%)	1,400 (83.0%)	1,750 (86.2%)
Smoking	6,467 (22.0%)	8,078 (38.5%)	665 (23.7%)	1,180 (36.4%)	288 (17.1%)	516 (25.4%)
ACEI or ARB	23,805 (81.2%)	14,777 (70.3%)	2,212 (78.9%)	2,346 (72.3%)	1,402 (83.1%)	1,489 (73.3%)
Aldosterone receptor antagonist	8,665 (29.5%)	4,979 (23.7%)	941 (33.5%)	834 (25.7%)	452 (26.8%)	458 (22.6%)
Angiotensin-receptor-neprilysin inhibitor	1,045 (3.6%)	318 (1.5%)	97 (3.5%)	49 (1.5%)	56 (3.3%)	20 (1.0%)
Antiarrhythmic	3,830 (13.1%)	3,196 (15.2%)	213 (7.5%)	309 (9.5%)	155 (9.2%)	193 (9.5%)
Non-dihydropyridine CCB	1,677 (5.7%)	1,874 (8.9%)	149 (5.3%)	224 (6.9%)	71 (4.2%)	133 (6.6%)
Loop diuretic	19,661 (67.0%)	14,695 (70.0%)	2,048 (73.0%)	2,404 (74.1%)	1,139 (67.5%)	1,426 (70.2%)
Beta blocker, any	26,485 (90.3%)	16,964 (80.8%)	2,575 (91.8%)	2,668 (82.2%)	1,512 (89.6%)	1,661 (81.8%)
Beta blocker, HFrEF guideline-directed	25,522 (87.0%)	15,926 (75.8%)	2,503 (89.2%)	2,558 (78.9%)	1,473 (87.3%)	1,587 (78.1%)
CRT-D	16,412 (56.0%)	11,009 (52.4%)	1,209 (43.1%)	1,292 (39.8%)	782 (46.4%)	935 (46.0%)
Total number of prescriptions	13.0 (6.0)	13.9 (7.1)	12.9 (6.2)	13.8 (7.3)	13.6 (6.6)	15.1 (8.2)
Outpatient visits prior to ICD	9.1 (9.0)	8.5 (9.5)	8.7 (8.9)	7.8 (8.6)	7.5 (8.2)	6.9 (8.1)

Based on data within the 12 months preceding implantable cardioverter-defibrillator implantation. All are *n* (%), mean (standard deviation) or median (25th, 75th percentile) unless otherwise specified. ACEI or ARB; angiotensin-converting enzyme inhibitor or aldosterone receptor blocker; CCB, calcium channel blocker; CRT-D, cardiac resynchronization therapy defibrillator; HFrEF, heart failure with reduced ejection fraction; ICD, implantable cardioverter-defibrillator.

^a^ Implantable cardioverter-defibrillator placement occurring during an unplanned hospitalization or within 90 days of a previous hospitalization as defined in the primary manuscript text.

**Table 3:** Modified Poisson regression models for association of race and ethnicity with peri-hospitalization implantable cardioverter defibrillator placement.

	Unadjusted	Age- and sex- adjusted	Age-, comorbidity- and SES- adjusted	Fully adjusted
	RR (95% CI)	RR (95% CI)	RR (95% CI)	RR (95% CI)
Black	1.29 (1.25, 1.32)	1.31 (1.28, 1.35)	1.18 (1.14, 1.22)	1.16 (1.12, 1.20)
Hispanic	1.31 (1.27, 1.35)	1.32 (1.28, 1.36)	1.09 (1.05, 1.13)	1.10 (1.06, 1.14)
Other	1.21 (1.15, 1.27)	1.22 (1.17, 1.29)	1.12 (1.06, 1.17)	1.09 (1.04, 1.15)
White	Referent	Referent	Referent	Referent
Female	—	1.03 (1.01, 1.05)	1.04 (1.02, 1.06)	1.05 (1.03, 1.07)

Fully adjusted models adjusted for age, sex, race and ethnicity, atrial fibrillation, anemia, cancer, chronic obstructive pulmonary disease, diabetes, chronic kidney disease, myocardial infarction, cerebral vascular disease, peripheral vascular disease, dementia, zip-code linked: population density, percent living in poverty, percent owner occupied housing, percent of population that is black, percent population hispanic, percent completing high school, medicaid dual eligibility, number of outpatient visits, number of prescriptions, the presence of guideline directed heart failure therapies (appropriate beta blockers, angiotensin-converting enzyme inhibitor, aldosterone receptor blocker, angiotensin-receptor-neprilysin inhibitor therapy, aldosterone receptor antagonists, loop diuretics, hydralazine and isosorbide dinitrate) other cardiovascular medications (digoxin, warfarin, antiplatelet agents, and non-dihydropyridine calcium channel blockers) and cardiac resynchronization therapy. SES, socioeconomic factors. Peri-hospitalization indicates implantable cardioverter-defibrillator placement occurring during an unplanned hospitalization or within 90 days of a previous hospitalization as defined in the primary manuscript text.

**Figure 2:**
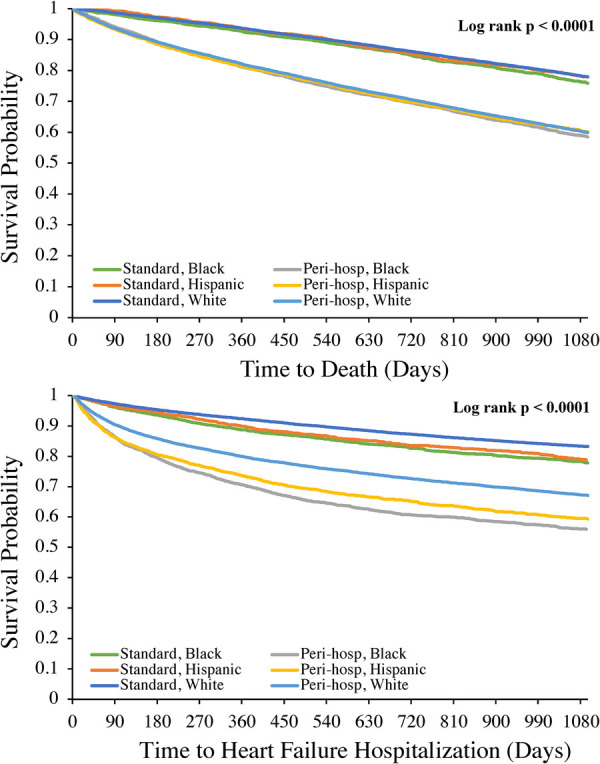
Kaplan-Meier analyses of mortality (top panel) and heart failure hospitalization (bottom panel) at 3 year follow-up after implantable cardioverter-defibrillator placement by race and ethnicity. Log rank *p* value was also <0.0001 for each outcome at 1-year follow-up.

**Figure 3:**
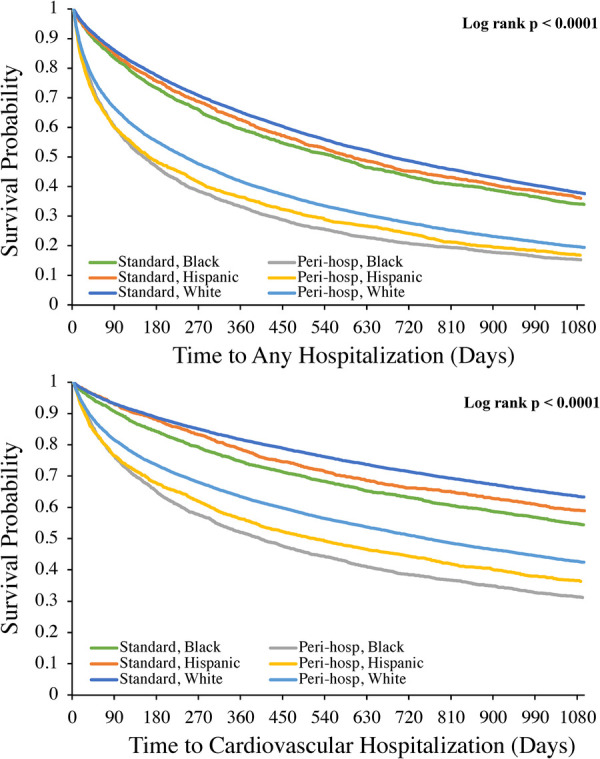
Kaplan-Meier analyses of all cause hospitalization (top panel) and cardiovascular hospitalization (bottom panel) at 3 year follow-up after implantable cardioverter-defibrillator placement by race and ethnicity. Log rank *p* value was also <0.0001 for each outcome at 1-year follow-up.

Kaplan-Meier analyses for 3-year follow-up by ICD placement status are shown in Figures 2, 3. Relative to “standard” planned ICD placement—defined as *not* occurring during an unplanned admission or within 90 days of a previous hospitalization—peri-hospitalization ICD placement was associated with worse mortality and hospitalization in every race and ethnicity group during long-term follow-up (Log rank *p* < 0.0001 for all outcomes). These differences emerged early in the follow-up period post-implantation (log-rank *p* < 0.0001 for 3 year; data not shown) and appeared to expand over the first 3 years. Mean follow-up time for the mortality outcome was 812 days.

For each hospitalization outcome among either ICD placement status, the highest incidence rates were among Black and Hispanic beneficiaries with peri-hospitalization ICD placement at both 1- and 3-year follow-up compared to White beneficiaries (Table 4). In fully adjusted Cox models, peri-hospitalization ICD placement remained associated with a significantly greater hazard of mortality, all-cause, HF and CV hospitalization post-implant within each race and ethnicity group at both 1- and 3-year follow-up (Table 4). At 3-year follow-up, there remained at least a 1.5× increased risk of death, 1.5× increased risk of all cause re-hospitalization, 1.7× increased risk of HF hospitalization and 1.5× increased risk of cardiovascular hospitalization for each group (Table 4) with peri-hospitalization placement. In pooled models (not stratified by race and ethnicity), there was no statistically significant difference in the associated higher risk with peri-hospitalization placement between race and ethnicity groups for any outcome (*p* = NS for race-ICD status interaction at both 1- and 3-year follow-up).

**Table 4:** Cox proportional hazard regression models for association of peri-hospitalization implantable cardioverter defibrillator placement with mortality and hospitalizations post-implant, by race and ethnicity.

	One year follow-up			Three year follow-up		
	Incidence rate per 1,000 person-years	Unadjusted	Fully adjusted	Incidence rate per 1,000 person-years	Unadjusted	Fully adjusted
		HR (95% CI)	HR (95% CI)		HR (95% CI)	HR (95% CI)
All-cause mortality						
Black						
Peri-Hospitalization	212.2	2.74 (2.33, 3.21)	1.93 (1.63, 2.29)	183.0	2.05 (1.85, 2.26)	1.52 (1.37, 1.69)
Standard	78.4	Referent	Referent	89.9	Referent	Referent
Hispanic						
Peri-hospitalization	212.2	3.16 (2.55, 3.92)	2.15 (1.70, 2.71)	175.9	2.17 (1.90, 2.47)	1.51 (1.31, 1.74)
Standard	67.3	Referent	Referent	81.3	Referent	Referent
Other						
Peri-hospitalization	198.5	2.67 (1.95, 3.65)	2.05 (1.46, 2.88)	187.7	1.96 (1.62, 2.35)	1.51 (1.23, 1.85)
Standard	75.1	Referent	Referent	96.2	Referent	Referent
White						
Peri-hospitalization	202.8	3.06 (2.89, 3.24)	2.05 (1.93, 2.18)	175.3	2.17 (2.10, 2.25)	1.55 (1.50, 1.61)
Standard	66.2	Referent	Referent	80.8	Referent	Referent
Any hospitalization						
Black						
Peri-hospitalization	1,321.5	2.23 (2.07, 2.40)	1.77 (1.64, 1.92)	940.7	1.98 (1.86, 2.11)	1.62 (1.51, 1.73)
Standard	548.0	Referent	Referent	418.2	Referent	Referent
Hispanic						
Peri-hospitalization	1,222.8	2.29 (2.07, 2.52)	1.73 (1.56, 1.93)	859.8	1.98 (1.83, 2.15)	1.56 (1.43, 1.71)
Standard	492.3	Referent	Referent	388.2	Referent	Referent
Other						
Peri-hospitalization	1,008.9	2.17 (1.86, 2.53)	1.66 (1.41, 1.96)	723.4	1.92 (1.69, 2.18)	1.55 (1.35, 1.78)
Standard	439.6	Referent	Referent	344.0	Referent	Referent
White						
Peri-hospitalization	1,014.7	2.14 (2.08, 2.20)	1.67 (1.63, 1.72)	731.4	1.87 (1.83, 1.92)	1.51 (1.47, 1.54)
Standard	450.0	Referent	Referent	362.4	Referent	Referent
Heart failure hospitalization						
Black						
Peri-hospitalization	373.6	3.01 (2.64, 3.43)	2.46 (2.14, 2.84)	239.5	2.51 (2.26, 2.78)	2.08 (1.86, 2.32)
Standard	121.7	Referent	Referent	89.9	Referent	Referent
Hispanic						
Peri-hospitalization	332.2	2.98 (2.50, 3.56)	2.17 (1.79, 2.63)	211.6	2.40 (2.10, 2.75)	1.83 (1.58, 2.12)
Standard	107.9	Referent	Referent	83.5	Referent	Referent
Other						
Peri-hospitalization	267.5	2.46 (1.87, 3.24)	1.69 (1.25, 2.28)	180.1	2.25 (1.82, 2.80)	1.70 (1.34, 2.15)
Standard	106.5	Referent	Referent	77.0	Referent	Referent
White						
Peri-hospitalization	239.4	2.86 (2.72, 3.02)	2.12 (2.00, 2.24)	155.3	2.31 (2.22, 2.41)	1.77 (1.70, 1.85)
Standard	81.6	Referent	Referent	64.6	Referent	Referent
Cardiovascular hospitalization						
Black						
Peri-hospitalization	721.2	2.30 (2.10, 2.52)	1.90 (1.72, 2.10)	500.1	2.06 (1.91, 2.22)	1.74 (1.60, 1.88)
Standard	302.5	Referent	Referent	224.8	Referent	Referent
Hispanic						
Peri-hospitalization	635.5	2.46 (2.17, 2.79)	1.89 (1.65, 2.17)	428.3	1.89 (1.89, 2.03)	1.67 (1.50, 1.86)
Standard	246.9	Referent	Referent	191.5	Referent	Referent
Other						
Peri-hospitalization	511.8	2.17 (1.78, 2.64)	1.57 (1.27, 1.95)	362.0	1.96 (1.67, 2.29)	1.50 (1.27, 1.78)
Standard	229.0	Referent	Referent	176.5	Referent	Referent
White						
Peri-hospitalization	498.5	2.31 (2.23, 2.39)	1.79 (1.72, 1.86)	345.1	2.00 (1.95, 2.06)	1.60 (1.56, 1.65)
Standard	209.1	Referent	Referent	163.8	Referent	Referent

Peri-hospitalization indicates implantable cardioverter-defibrillator placement occurring during an unplanned hospitalization or within 90 days of a previous hospitalization as defined in the primary manuscript text. Fully adjusted models included all covariates in the modified Poisson regression models except that these Cox models also adjusted for peri-hospitalization ICD placement.

## Discussion

In this study, we assessed potential racial and ethnic differences in “peri-hospitalization” ICD placement and whether such differences were associated with disparate post-implant outcomes.

We found a higher proportion of peri-hospitalization ICD placements among Black and Hispanic than White beneficiaries that persisted throughout the study period. These differences were not explained by sociodemographic or clinical factors. We also found that mortality and hospitalization rates were higher after peri-hospitalization versus “standard” ICD placement among all racial and ethnic groups and that this increased risk did not differ significantly between groups.

Previous studies have suggested that the setting in which ICD placement occurs for primary prevention of SCD in HFrEF is relevant to whether the desired benefit is observed. One study in approximately 23,000 older Medicare beneficiaries with HF deemed eligible for ICD placement for primary prevention found that placement during an acute hospitalization was not associated with improved mortality compared to groups not receiving an ICD (4). Another study of over 80,000 Medicare beneficiaries who underwent ICD placement for primary prevention found that placement during an acute HF hospitalization was associated with higher mortality and all cause re-hospitalization within 90 days (3). Despite these significant contributions to the literature, prior to our study, possible race and ethnicity differences in setting of ICD placement and potential associations with long term outcomes had not been well described. Our study contributes significantly to the literature by investigating how those who receive ICDs during or early after any hospitalization may fare differently with regards to important outcomes within and across race and ethnicity groups.

Suboptimal representation of racial and ethnic minority populations in seminal clinical trials demonstrating mortality benefit with primary prevention ICD placement for HFrEF have spurred questions about whether all racial and ethnic groups derive similar benefit from ICD therapy. These questions are complicated by the fact that race and ethnicity primarily reflect a social construct which often is a surrogate for other sociodemographic factors and social determinants amongst other factors, rather than being rooted in biology (15). Despite worse overall HF mortality among Black populations, multiple studies have demonstrated that Black and other ethnic minority populations derive similar mortality benefit from primary prevention ICD placement as White populations (11–13). Our data suggests that attention to timing of ICD placement during or close to a recent hospitalization should be an important consideration in efforts to derive the greatest benefit from ICD therapy. Furthermore, whether addressing racial and ethnic differences in setting of placement could contribute to efforts to reduce some disparate HF outcomes is a consideration that deserves close inspection. One potential explanation for the observed differences in outcomes is that placement during or soon after hospitalization is a marker of inadequate optimization or greater illness at the time of placement, whether that be from the standpoint of HF or other clinical conditions. Just as optimization of guideline-directed HF therapy prior to ICD placement for primary prevention is considered essential, having clinically stable HF and other comorbid conditions for at least a period of 3 months may be another important consideration to derive the greatest real-world effectiveness from ICD placement. Waiting an adequate time after hospitalization may not only reduce complications but may be important for ensuring that overall prognosis related to co-morbidities is sufficient such that ICD placement confers meaningful survival benefit. Our findings are supported by previous data suggesting that Medicare beneficiaries without a HF hospitalization within a year preceding ICD placement had similar mortality as observed in major primary prevention ICD therapy trials, whereas ≥1 HF hospitalization preceding placement was associated with worse mortality (16).

Previous investigators have argued that current paradigms are not sufficient to optimally predict who will most likely derive benefit from ICD placement for primary prevention in HFrEF when weighed against an overall low sudden cardiac death risk with contemporary HF medical therapies in some individuals or against the presence of significant competing risks for poorer outcomes in others (10, 17). At the same time, racial and ethnic differences in ICD placement for eligible populations with HFrEF have been well-documented with some previous data focused specifically on these differences during HF hospitalization (8, 18). In this context, potential explanations for the differences in frequency of peri-hospitalization ICD placement deserve attention, especially given that these differences by race and ethnicity did not appear to be explained by socioeconomic or clinic factors. It is reasonable to consider that these differences in frequency could reflect implicit (or explicit) bias by some providers that certain race and ethnicity groups will be less medically adherent, including to follow-up after hospitalization (19). Alternatively, our findings may reflect an unintended downstream impact of past data which importantly created greater awareness of racial and ethnic differences in ICD placement, but did so through a focus on ICD placement *during hospitalization*. One also cannot exclude the influence of patient and provider preferences, possibly based on factors related to access to care that are not well-reflected by socioeconomic and health care utilization markers. Undoubtedly, race and ethnicity-based differences in the offering of ICD therapy during hospitalization to patients meeting guideline-based indications are important to document, as they are a contributor to the overall racial and ethnic disparities in ICD placement. Perhaps, an adjusted paradigm is needed that focuses both on assuring that racial and ethnic disparities in ICD placement are eliminated, in addition to reducing differences in the optimal timing and setting of ICD placement. We note that our findings suggest that the peri-hospitalization placement was not a primary driver of differences in mortality and hospitalizations after ICD placement between racial and ethnic groups. Nevertheless, an approach accounting for the timing and setting of ICD placement is worth consideration in the goal of improving outcomes within and across racial and ethnic groups.

This study has important limitations. We utilized *International Classification of Diseases* diagnosis codes to identify co-morbid conditions and types of hospitalizations. Although we specified diagnoses to focus on a population with HFrEF receiving an ICD for primary prevention, there is no ejection fraction data available in Medicare data, which would be the most optimal way to identify this population. However, *International Classification of Diseases* codes for HF have demonstrated good positive predictive value in validation studies (20, 21). In addition, we excluded possible diagnoses that could be indications for ICD placement as secondary prevention. We incorporated data linked to the five-digit zip code of beneficiaries, a commonly used geographic unit in epidemiologic research but that nevertheless is not as precise as more granular socioeconomic data. We note a low proportion of beneficiaries on Angiotensin-receptor-neprilysin inhibitor therapy which was newly approved during the study period in 2015. Although the less-than-ideal prescribing of these agents is well documented despite their effectiveness (22, 23), future study will be needed to investigate whether higher uptake of these agents would affect the study outcomes. Due to limitations related to cohort size, we focused our analyses on the largest available racial and ethnic groups in the Medicare data, including Black, Hispanic, and White populations. Thus, our findings may not be generalizable to populations of other race and ethnicities.

## Conclusion

ICD implantation occurring during the peri-hospitalization period was associated with worse prognosis and occurred at higher rates among Black and Hispanic compared to White Medicare beneficiaries with HF during the period under study. The risk associated with peri-hospitalization ICD placement did not differ by race and ethnicity. Future paradigms aimed at enhancing real-world effectiveness of ICD therapy and addressing disparate outcomes should consider timing and setting of ICD placement in HFrEF patients who otherwise meet guideline eligibility.

## Data Availability

The data analyzed in this study is subject to the following licenses/restrictions: the data are available to researchers by request to and through data use agreements with the Centers for Medicare and Medicaid Services (CMS). The data cannot be shared without permission as per agreement with CMS. Requests to access these datasets should be directed to https://resdac.org/.
