# Supplementary material for: Racial differences in setting of implantable cardioverter-defibrillator placement in older adults with heart failure and association with disparate post-implant outcomes
Source: Front Cardiovasc Med. 2023 Sep 1;10:1197353. doi: 10.3389/fcvm.2023.1197353 (PMC10505431; doi:10.3389/fcvm.2023.1197353)
Supplement: Supplementary file 1 [file Table1.docx]

**Supplemental Material**

**Contents**

**eTable 1: International Classification of Diseases Diagnosis Codes and Current Procedural Terminology Codes Used for Exclusion Criteria**

**eTable 1: International Classification of Diseases Diagnosis Codes and Current Procedural Terminology Codes Used for Exclusion Criteria**

| **Exclusion Criteria** | **International Classification of Diseases Code (ICD9)** | **International Classification of Diseases Code (ICD10)** | **Healthcare Common Procedure Coding System and Current Procedural Terminology Code** |
| --- | --- | --- | --- |
| **Cardiac Arrest, Ventricular Tachycardia, Ventricular Fibrillation, Ventricular Flutter** | 427.1, 427.5, 427.41, 427.42 | I46.9, I49.01, I49.02 | Not applicable |
| **Myocardial Infarction** | 410.x | I21.0x, I21.1x, I21.3, I21.4, I22.0, I22.1, I22.2, I22.8, I22.9 | Not applicable |
| **Revascularization** | Not applicable | Not applicable | 33510-33514, 33516-33523, 33533-33536, 33572, 92920-92921, 92924-92925,92933- 92934, 92937-92938, 92941, 92943-92944, 92982, 92984, 92995-92996, 93540, 93564, 93570, C9602-C9608, S2205-S2209 |
| **Arrhythmogenic Syndrome (Brugada, Catecholaminergic polymorphic ventricular tachycardia, Long QT Syndrome)** | 746.89, 427.1, 426.82, | I49.8, I47.2, I45.81 | Not applicable |
| **Implantable Cardioverter-Defibrillator Device replacement (existing ICD device prior to ICD implantation)** | V45.02 | Z95.810 | Not applicable |
| **Permanent Pacemaker Indication** | 426.x, 427.81, 427.89 | I44.x, I49.5, I49.8 | Not applicable |
